# Changes of macular pigment optical density in elderly eyes: a longitudinal analysis from the MARS study

**DOI:** 10.1186/s40942-016-0039-6

**Published:** 2016-06-01

**Authors:** Verena Meyer zu Westrup, Martha Dietzel, Meike Zeimer, Daniel Pauleikhoff, Hans-Werner Hense

**Affiliations:** 1 Institute of Epidemiology and Social Medicine, Medical Faculty, Westfälische Wilhelms University, Albert-Schweitzer-Campus 1, D 3, 48149 Münster, Germany; 2 Ophthalmology Department, St. Franziskus Hospital, Münster, Germany

**Keywords:** Age-related macular degeneration, Macular pigment optical density, Two-wavelength autofluorescence, Prospective study

## Abstract

**Background:**

Macular pigment (MP) has been related to the occurrence of age related macular degeneration (AMD). We investigated prospectively in eyes of elderly individuals how magnitude and spatial distribution of MP had changed after 4 years.

**Methods:**

The study included 380 eyes from 237 participants of the Münster Ageing and Retina Study cohort which were free of advanced stages of AMD. MP optical density (MPOD) was measured in density units (D.U.) at eccentricities of 0.25°, 0.5°, 1.0° and 2.0° from the fovea using dual-wavelength autofluorescence; ring-like MP distributions were identified from MP density profiles. Changes were assessed with mixed linear models.

**Results:**

The study participants’ mean age at baseline was 70.5 years. Early AMD was present in 150 study eyes (39.5 %) and a ring-like distribution of MPOD was found in 87 study eyes (22.9 %). After a median follow-up time of 3.96 years, the MPOD averaged over all eyes was slightly raised at the central fovea (from 0.658 to 0.670 D.U. (relative change +1.8 %), p = 0.08) and most markedly at 2.0° (from 0.157 to 0.172 D.U. (+9.5 %), p < 0.001). Multivariate analyses, adjusting for sex, body mass and carotenoid supplement intake, revealed that MPOD increments, at any distance from the fovea, were slightly less pronounced in older eyes. Serum concentrations of lutein at follow-up, presumably reflecting recent intake of antioxidant supplements, raised MPOD levels significantly at 1.0° and 2.0° (both p < 0.01) but not in the central fovea. Early AMD at baseline and ring-like MPOD distribution did not significantly impact on MPOD changes over time. A ring-like spatial distribution of MPOD persisted in over 80 % of the affected eyes.

**Conclusions:**

Overall, the magnitude and spatial arrangement of MPOD was remarkably stable over time in elderly eyes. Significant MPOD rises in perifoveal regions probably indicate effects of lutein containing supplements. The persistence of ring-like MPOD distributions over time seems to suggest their determination by anatomical structures.

**Electronic supplementary material:**

The online version of this article (doi:10.1186/s40942-016-0039-6) contains supplementary material, which is available to authorized users.

## Background

Recent research has shown that the occurrence and progression of age-related macular degeneration (AMD), the leading cause of legal blindness among the elderly population in industrialized countries [1], is mainly influenced by demographic, environmental and genetic factors [2–4]. The level of macular pigment in the central retina is thought to also have an impact on AMD, however, the precise roles and mechanisms of this process are not yet clearly understood [5, 6]. Commonly, lutein meso-zeaxanthin and zeaxanthin, known to accumulate in the macula as macular pigment (MP) [7] and having antioxidant and light-screening properties for short wavelengths, are hypothesized to protect the eye against the development of various degenerative retinal diseases, including AMD [8].

There is presently some debate as to whether a loss of MP is the result of progressed AMD or whether it is a cause [9]. It seems conceivable that degenerative processes cause impairments in transport and storage of lutein and zeaxanthin resulting in decreased MP in the retina [5]. On the other hand, a reduced availability and potentially also storability of carotenoids in the retina may independently lead to the degeneration of functional retinal tissue. Zeimer et al. [10] demonstrated that, after oral supplementation with lutein and zeaxanthin, an increase in MPOD can be detected only in areas where MPOD had been measurable before in persons with macular telangiectasia. Supplementation did not produce an increase in areas where MPOD had been lacking at baseline. These results are compatible with the hypothesis that MP is stored in retinal tissue structures that are characteristic of an individual and that determine also the spatial patterns of MPOD distribution [11]: supplementation can therefore increase MPOD levels only where such storage facilities were pre-existing. Interestingly, ring-like distributions of MPOD appear not to be affected by supplementation of lutein/zeaxanthin [12]. According to Tariq et al. [13] such ring-like patterns are highly inheritable and constant in individuals. These results support the idea that genetically determined structural components are responsible for the storability and availability of MP in the macula and that successful supplementation with lutein or zeaxanthin always depends on macular microanatomy. Our recent study using optical coherence tomography supports such a concept [11].

It is also not entirely clear in which tissue compartments the MP is stored. It is known to primarily accumulate in the inner plexiform layer and the long cone receptor axons (Henle’s fibres) [14], and more recent studies have attributed Müller cells with the trafficking and storage of MP within the retina [15]. Thus, it has been hypothesized that low levels or loss of MP may be causal to the dysfunction or destruction of Müller cells. The latter may be associated with the ring-like MPOD distribution that is found in a certain proportion of retinae [11].

To further elucidate these relationships, we investigated whether the contents and the distribution patterns of MP change over time with the ageing of elderly individuals [16]. The measurement of its optical density (MPOD) served as a non-invasive method of quantitative MP assessment and it was prospectively analysed in a cohort of elderly Caucasian individuals who were followed for 4 years.

## Methods

### Study subjects

Data were obtained from participants of the Münster Ageing and Retina Study (MARS), a longitudinal study designed to identify medical, environmental and genetic factors with implications for the pathogenesis and progression of early AMD [17, 18]. Eligibility criteria and examination protocols have been described in detail before [4, 17, 19]. In brief, patients were self-selected ophthalmology patients residing in the Münster region in North-Western Germany. Patients had to be aged between 60 and 80 years by the time the study started, had to have early AMD in at least one eye and no contraindications to pupil dilatation, no advanced cataracts or other factors impeding clear visualization of the central retina. Additionally, a convenience sample consisting of spouses or friends of study participants and of other volunteers that were free of AMD, were included as a comparison group. All participants were of Caucasian origin.

The cohort baseline examinations (MARS I) of 1060 participants took place from June 2001 to October 2003. The first cohort follow-up (MARS II) was carried out between November 2003 and August 2006 with 828 participants attending (85.5 % of those eligible), of whom 722 had gradable fundus photographs in both eyes. Between 2007 and 2009, after a median time of 3.96 years, we re-examined 492 participants (MARS III, participation 75.1 % of those eligible).

At each study visit, participants were interviewed by trained staff members using mostly identical, standardized questionnaires. Detailed information was obtained on demographic characteristics, smoking, lifestyle, medical history, and the current use of medications and vitamin supplements, in particular those containing the carotenoids L and/or Z. Ophthalmological examinations were performed to determine the best corrected visual acuity (Early Treatment Diabetic Retinopathy Study charts) and by using slit-lamp microscopy. The spherical equivalent refractive error in each eye and the number of pseudophakic eyes were documented.

Stereoscopic digital fundus photography was performed at an angle of 30° in both eyes, centred on the fovea. The grading of AMD stages was performed according to the Rotterdam Classification System [20] as described in detail before [2, 18]. The range of AMD signs was stratified into five severity stages: stage 0, no sign of AMD or hard drusen (<63 μm) only; stage 1, soft, distinct drusen (≥63 μm) only, or pigment epithelium changes only, no soft drusen (≥63 μm); stage 2, soft, indistinct drusen (≥125 μm) only, or soft distinct drusen (≥63 μm) with pigment epithelium changes; stage 3, soft, indistinct drusen (≥125 μm) with pigment epithelium changes; and stage 4, atrophic or neovascular AMD. Eyes were classified as having no AMD (stage 0 or 1), early AMD (stages 2–3) or late stage AMD (stage 4). All baseline and follow-up ophthalmologic examinations were conducted by specifically trained medical staff whose performance was regularly certified. The follow-up visits involved the same examinations as the baseline (MARS I) visits [16, 19] plus additional components such as, e.g., MPOD measurements which were included in MARS II and MARS III.

AMD was diagnosed in eyes with dilated pupils based on presence and severity of retinal lesions.

### MPOD measurements

The two-wavelength autofluorescence (AF) method for measuring MPOD has been previously described [7, 21–24]. Briefly, it is based on the AF of lipofuscin, which is present in the retinal pigment epithelium (RPE) cells. Lipofuscin can be excited in vivo between 400 and 580 nm to emit its fluorescence in the 500–800 nm spectral range, whereas MP absorbs blue-light for wavelengths shorter than 550 nm, with a peak absorbance of 460 nm. In the fovea, excitation light within the absorption range of MP is partially absorbed by the carotenoids, resulting in an area of reduced fluorescence. In order to measure the MPOD, the dual-wavelength approach of the AF method compares results from two excitation wavelengths that are differentially absorbed by the MP. Therefore, the dual-wavelength technique takes account of the non-uniform distribution of lipofuscin in the RPE but assumes that the shape of the excitation spectrum is constant over the macular area [19].

In our analyses, quantitative imaging was performed using a retinal angiograph (Heidelberg Retina Angiograph HRA 1; Heidelberg Engineering, Heidelberg, Germany), modified for the measurement of macular pigment. Excitation wavelengths used were 488 nm (well absorbed by MP) and 514 nm (minimally absorbed by MP). This method has been used in clinical studies and was described in detail before [12]. MPOD measurements were added to the examination procedures during MARS II and were subsequently also obtained in the participants of MARS III. All MPOD measurements were performed by at least two trained investigators using the same testing device and protocol throughout. Subjects with AF images of inadequate quality (n = 83) (most commonly due to insufficient fixation by the study subjects) were excluded as described before [19].

### MP optical density profile and ring-like structure

In consistency with our previous research on spatial distribution of MP we distinguished between eyes with and without ring-like MPOD structures and a third group of eyes with an intermediate structure [11, 16, 19]. Measurement of spatial distribution has been described previously in detail. In brief, we analyzed the MP optical density maps and the radial density profiles. The latter were generated and graphically displayed by plotting the mean MPOD values that were calculated for each radius around the fovea in distances of up to 8°, displaying a profile of grey scale values comparable to a cross-sectional cut through the foveola. In eyes where the fovea could not be defined automatically as the center of the Gaussian distribution fitted to the MPOD map, the center of the fovea was defined manually (Additional file 1: Figure S1).

Ring-like structures were defined as density profiles showing bimodal patterns consisting of a central peak of MPOD followed by a decline and a secondary peak of increased density on the slope of the profile. In those cases where the distribution of MPOD showed neither a strictly monotonic decline from the foveola to the periphery nor an explicit ring-like pattern, such eyes were labelled as having “intermediate distributions” and were treated as a third group of eyes besides those distinctively categorized as with or without ring-like structures [16].

For this study, we present the mean MP optical density averaged along an annulus with retinal eccentricity of 0.25°, 0.5°, 1.0° and 2.0° degrees and width of one pixel each. MPOD results are reported in dimensionless density units (D.U.).

### Study participants

**Fig. 1:**
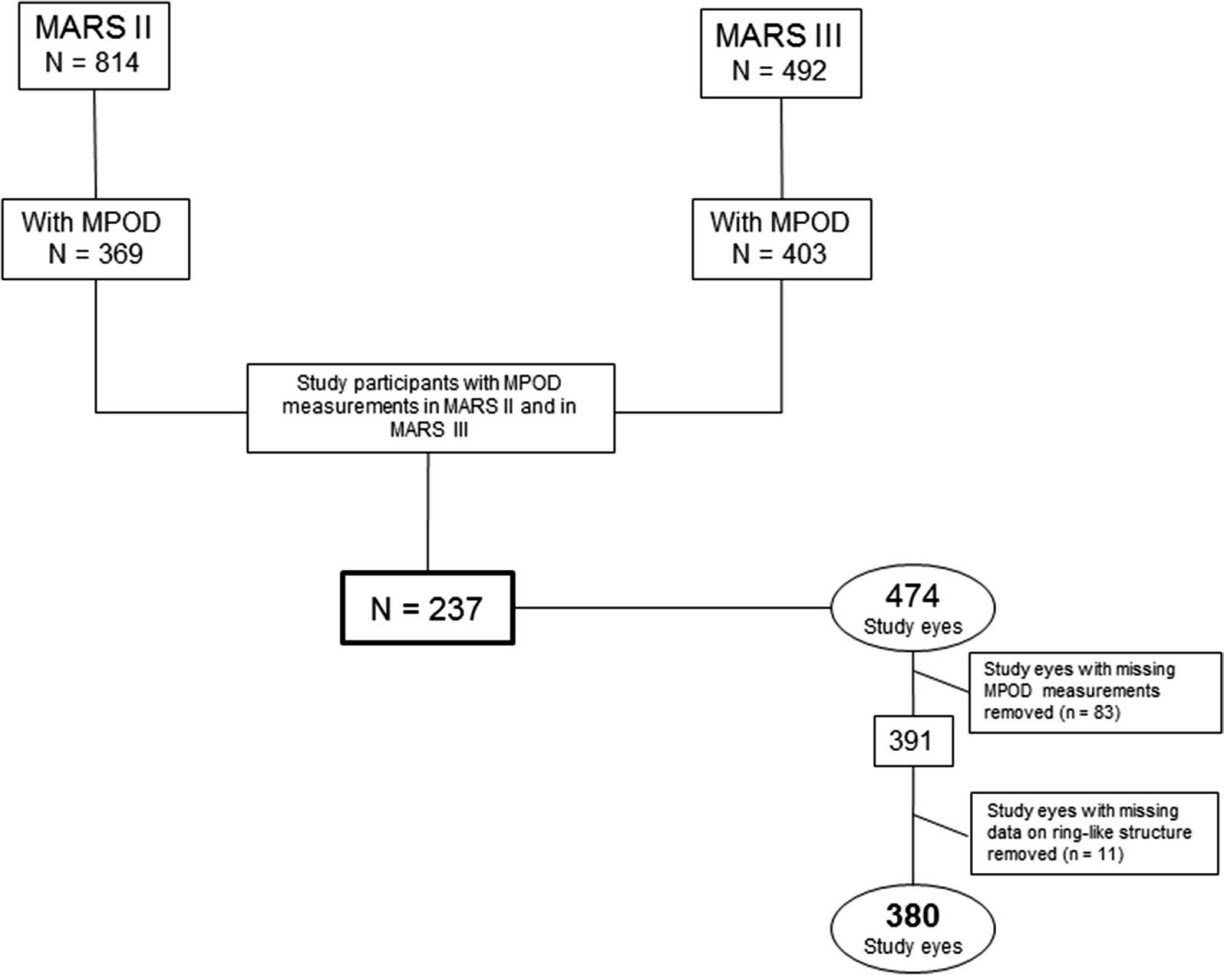
Flowchart to visualize the recruitment of suitable study subjects

MPOD measurements were included as an expansion of the study protocol during the second half of MARS II: this defined the start of the MPOD sub-study reported herein. To better visualize the selection process see the flowchart in Fig. 1. There were 237 participants—providing 474 single eyes for the study—who had MPOD measured in both MARS II and in MARS III, and gradable fundus photographs in at least one eye. In addition, participants were included in the study base only if they had a complete data set for potential confounders, that is, for age, sex, smoking, and body mass index (BMI) as measured at MARS II, and for serum concentrations of lutein and zeaxanthin as measured at MARS III. MPOD measurements of sufficient quality and data on the presence or absence of a ring-like structure were available in 380 of the initial 474 study eyes which formed the data base for the progression analyses presented in this report.

We used self-reports on the use of lutein and zeaxanthin (L/Z) containing vitamin supplements. These reports are highly variable due to the availability of an enormous amount of different preparations sold over the counter. Additionally, serum concentrations of lutein and zeaxanthin measured at the follow-up visit of MARS III helped to account for unmeasured lutein and zeaxanthin supplies at follow-up.

### Statistical analysis

Unadjusted comparisons were made using two-sided paired t-tests for the comparison of mean MPOD values at baseline and at follow-up. Changes of MPOD over time (ΔMPOD) were calculated by subtracting values measured in MARS III from those measured at baseline in MARS II. Multivariate regression methods were applied to model ΔMPOD at 0.25°, 0.5°, 1.0° and 2.0° eccentricities in order to assess the impact of age at baseline, serum lutein/zeaxanthin levels, presence of AMD and presence of ring-like structure accounting for sex, BMI and self-reported intake of L/Z-containing supplements. In sensitivity analyses, pseudophakia and spherical equivalent were additionally included in the models. As smoking prevalence was extremely low among the aged participants of this study, we did not include smoking in the models. To account for the interrelation between the paired eyes in the study sample, we applied linear mixed regression modelling. In addition, we assessed if the presence of ring-like or intermediate structures changed between the two measurements. All analyses were performed using the Statistical Analysis System (SAS, version 9.4 for Windows, SAS Institute Inc., Cary, NC).

## Results

The 380 study eyes originated from 237 individuals who were on average 70.5 years old at the start of the prospective follow-up. About two-thirds of the study eyes were from female participants and roughly one-third showed signs of early AMD while ring-like MPOD structures were found in about every fourth study eye (Table 1). The mean body mass index of the study participants was 27.0 (standard deviation 4.2). Of note, current smoking was very uncommon in this aged study group (only 3 active smokers) and therefore not further considered in the following analyses.

**Table 1:** Baseline characteristics of the study

Number of study participants	237
Age at MARS II (years)	
Mean	70.5
Standard deviation	4.8
Number of study eyes	380 (100 %)
Number of eyes from females	244 (64.2 %)
Eyes with	
Early AMD	150 (39.5 %)
No AMD	230 (60.5 %)
MPOD distribution	
Ring-like	87 (22.9 %)
Intermediate	69 (18.2 %)
Monotonically decreasing	224 (58.9 %)
Follow-up time (years)	
Median	3.96
Interquartile range	0.12
Minimum	2.94
Maximum	5.26

*MPOD* macular pigment optical density

Table 2 shows that after a median follow-up time of 3.96 years, the mean MPOD values at 0.25°, summarizing all eyes, was only slightly raised (+0.012 D.U., a 1.8 % relative increase over the baseline value; p = 0.08). The MPOD increment was also consistently low further away from the fovea, ranging from +0.011 to +0.015 D.U. (all p < 0.05). In relative terms, however, the most prominent and highly significant rise was observed at 2.0° with +9.6 % (p < 0.001). More detailed analyses, considering quintiles of MPOD change by location, confirmed this finding (Additional file 1: Figure S1).

**Table 2:** Macular pigment optical density (MPOD, in density units D.U.) in the MARS II and MARS III examinations and their differences (ΔMPOD)

	MPOD MARS II	MPOD MARS III	ΔMPOD	*p* value
	Mean (SEM)	Mean (SEM)	Mean (SEM)	
At 0.25°	0.658 (0.010)	0.670 (0.010)	0.012 (0.008)	0.0814
At 0.50°	0.568 (0.010)	0.583 (0.010)	0.014 (0.006)	0.0138
At 1.0°	0.449 (0.008)	0.461 (0.008)	0.011 (0.005)	0.0196
At 2.0°	0.157 (0.004)	0.172 (0.004)	0.015 (0.002)	<0.0001

Multivariate analyses (Table 3), adjusted for sex, body mass index, carotenoid supplementation, pseudophakia and spherical equivalent, revealed that older age at baseline was associated with statistically significant, reduced MPOD changes at 1.0° (−0.020 D.U. per 5 year older age, p > 0.001) and 2.0° (−0.0075 D.U. and p < 0.01, respectively); this effect was not present in the central regions of the retina. Lutein serum concentrations were directly related to MPOD increases; of note, these increments over time were detected exclusively at 1.0° (p < 0.01) and 2.0° (p < 0.001). The serum levels of zeaxanthin showed no association with MPOD. Furthermore, early AMD at baseline appeared unrelated to MPOD changes over time. Likewise, the presence of a ring-like MPOD distribution was not associated with significant changes of MPOD over time.

**Table 3:** Predictors of the change of MPOD (ΔMPOD) after a median follow-up of 3.96 years; the results are presented as regression coefficients (β) from multivariate regression models

	Change of MPOD (ΔMPOD)*							
	At 0.25°		At 0.5°		At 1.0°		At 2.0°	
Predictors	β	p value	β	p value	β	p value	β	p value
Age (per 5 years)	−0.019	0.05	−0.015	0.10	−0.020	<0.001	−0.0075	0.01
Log serum lutein [microg/ml] at follow-up	−0.008	0.69	+0.013	0.41	+0.016	<0.01	+0.023	<0.001
Log serum zeaxanthin [microg/ml] at follow-up	−0.01	0.73	−0.009	0.70	−0.006	0.74	−0.004	0.67
AMD (vs. none) at baseline	−0.006	0.75	−0.010	0.51	−0.007	0.55	+0.002	0.86
MPOD Ring (vs. none) at baseline	+0.024	0.24	+0.009	0.57	+0.013	0.31	+0.013	0.06

* Adjusted—in addition to variables in table—for sex, body mass index and carotenoid supplementation, pseudophakia and spherical equivalent

**Fig. 2:**
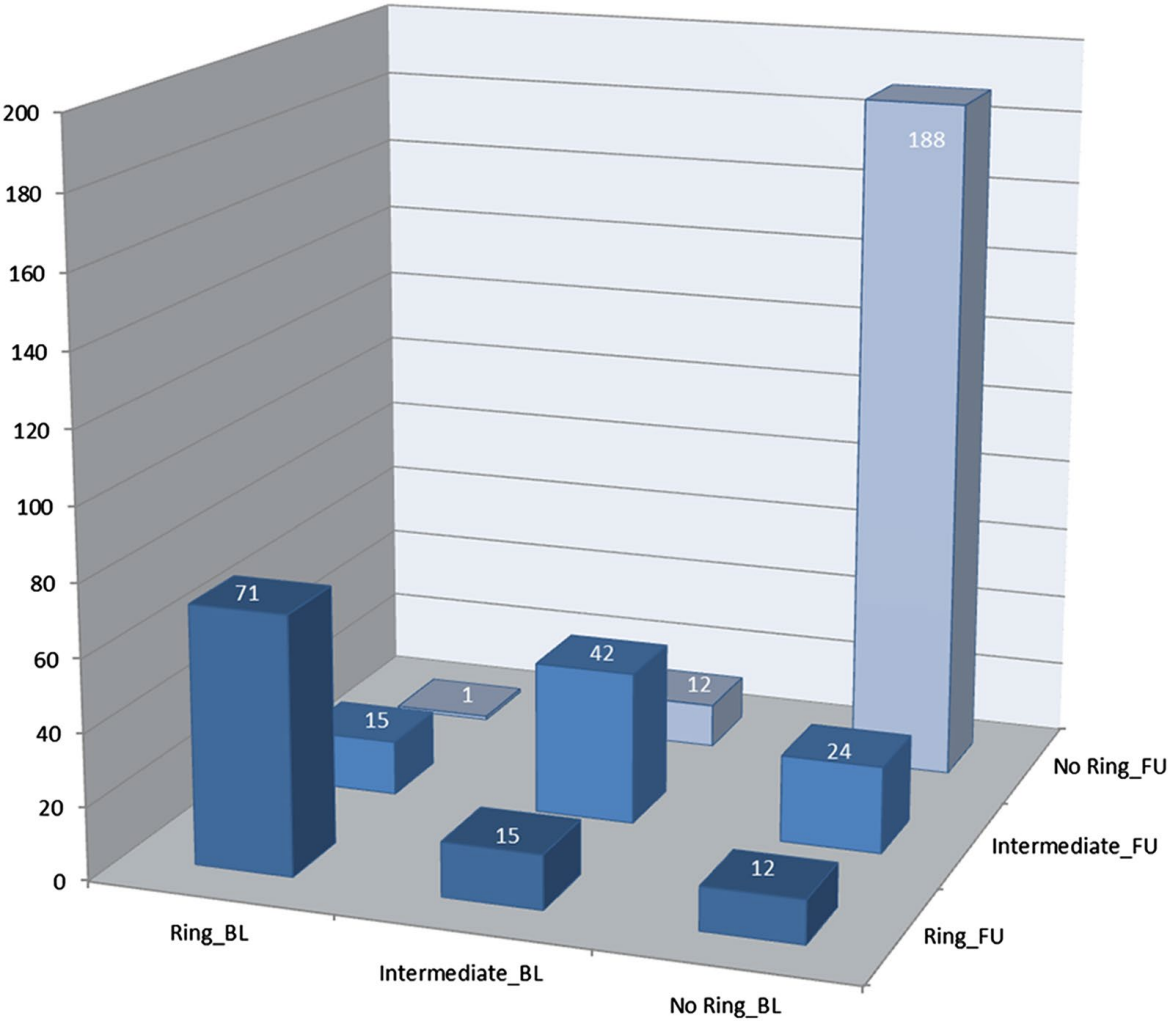
Cross-classification of study eyes according to spatial distribution patterns of MPOD: comparison of baseline and follow-up examinations. Ring_BL = ring-like MPOD distribution at baseline. Intermediate_BL = intermediate MPOD distribution pattern at baseline. No Ring_BL = monotonously decreasing MPOD distribution at baseline. Ring_FU = ring-like MPOD distribution at 3.96 years follow-up. Intermediate_FU = intermediate MPOD distribution pattern at 3.96 years follow-up. No Ring_FU = monotonously decreasing MPOD distribution at 3.96 years follow-up

Distribution patterns of MPOD persisted in 301 of the 380 eyes (79.2 %) during the study period (Fig. 2). Fifteen (17 %) of the 87 eyes with ring-like MPOD were re-classified as intermediate, and only 12 out of 224 (5.4 %) with no sign of a ring-like or intermediate MPOD distribution at baseline were now classified as ‘incident’ rings.

## Discussion

In this prospective study of elderly eyes, the levels of macular pigment increased only mildly (between 1.8 and 9.5 % relative change) over time and the spatial distribution was fairly stable. The MPOD increments were lower in eyes of older study participants, but this was statistically significant only at distances of 1.0° and 2.0° from the centre. Counterbalancing this was the effect of lutein in blood, presumably reflecting recent L/Z containing supplement use, raising MPOD levels also mainly in the perifoveal regions. The presence of early AMD or ring-like distributions at baseline had no obvious impact on MPOD alterations over time.

This is to our knowledge the first prospective observational study measuring MPOD changes in a cohort of elderly individuals who were not submitted to a study protocol involving systematic supplementation. The study cohort was reassessed after an average period of 4 years. We aimed to elucidate the process that may, at least in parts, reflect the ‘natural course’ of individual MPOD levels and distributions in elderly eyes due to ageing, environmental factors and disease. This study was observational and non-interventional by nature, permitting study individuals to start or stop the use of supplementary prescriptions indistinctively. The self-reported utilization of lutein and/or zeaxanthin containing supplements by study participants turned out to be highly variable probably due to recall errors. Supplements containing lutein and zeaxanthin in markedly varying concentrations are sold in Germany over the counter in pharmacies, drug stores and supermarkets—and their numbers count in hundreds. Therefore, the elderly participants could neither precisely recall brand names nor strengths of the supplement or duration of its intake. To corroborate the self-reported supplement use we measured the concentrations of lutein and zeaxanthin in serum at the time of the follow-up examination assuming that these concentrations more objectively reflected the individual situation with regard to recent supply with lutein and zeaxanthin. We observed that, even after controlling for self-reported supplement use at baseline and at follow-up, serum lutein concentrations were still significantly related to higher MPOD levels in the perifoveal region. By contrast, no MPOD changes were detected in the central foveal areas. These findings are in line with evidence showing lutein is predominantly stored in the perifoveal area [25] while zeaxanthin is stored in the fovea where it is presumably more stable and less easily influenced by supplementation [5] as also confirmed by a lack of associations with serum zeaxanthin in our study. This appears to also confirm previous reports postulating that the anatomical structure of the fovea plays an important role in the way MP is distributed [26]. As suggested before [11], the rather stable central load of MP is presumably attributable to the xanthophyll binding Müller cell cones, while in the periphery the cone axons store the MP. It appears that raised serum lutein is more easily integrated in the peripheral axon membranes than in the Müller cones, probably because zeaxanthin dominates capacities in the latter. It would be interesting to determine whether this observation is entirely attributable to capacity of storage, micro-anatomical changes such as dislocations, crushing or squeezing of cell complexes, or whether degenerative processes influence the fluctuation of MPOD.

Of note, the average net change of MPOD over 4 years was, despite being statistically significant at the more peripheral eccentricities, quantitatively small. In an attempt to disentangle the influential factors in this process, we found a tendency of old eyes to accumulate less MP in the retinal periphery. In the overall summary analyses of all eyes, this was counterbalanced by the MPOD rise in the same region attributable to higher lutein serum concentrations. It appears that the utilization of MP containing supplements which was rising with age was leading to the positive net MPOD balance reported in Table 2.

Interestingly, and in line with previous findings [11, 13], the presence and magnitude of ring-like distributions of MPOD remained fairly stable over time. This supports the view that the spatial MPOD patterns are individually rather stable features and that they derive rather from anatomical and/or genetic predetermination than from the influence of lifestyles, age or the availability of carotenoids. Nevertheless, we observed variations in that some eyes with a ring were classified at follow-up as intermediate (15/87) while some appeared as ‘incident’ ring structures. This variability may potentially be attributable to the methods of classification of ring-like distributions from density profiles. These are dependent on the precise identification of the fovea center. However, the same technical equipment and the same trained observers carried out the analyses in this prospective study. The impact of surgical interventions (e.g., cataract removal) which may have improved the image quality between the first and the second examination was ruled out as a major biasing factor in sensitivity analyses.

Our finding that the presence of early AMD at baseline was prospectively unassociated with MPOD change seems to indicate that the onset of AMD is not accompanied by a concomitant marked loss of MP.

Previous studies suggested that the reasons for lower MPOD levels are female sex, smoking, ethnicity and older age [27]. In this prospective study sex was unrelated to MPOD change over time and the impact of age was only very modest; the impact of smoking could not be assessed due to the low number of current smokers, and all participants were Caucasian. Thus, it may appear that MPOD levels and distribution in well-nourished elderly, avoiding nutritional depletion, is a stable and well conserved feature of the retina.

The strength of the presented report lies in its prospective design and the fact that measurements were repeated with consistent quality on a large number of eyes. As a major shortcoming we emphasize the difficulty of precisely and validly ascertaining the intake of supplements containing macular pigment components. Lutein levels in serum helped to account for this problem and we suppose that these measurements probably captured most of the influence of recent supplement utilization.

## Conclusion

In conclusion, MPOD was rather stable in levels and spatial arrangement over time in ageing eyes. Significant MPOD rises predominating in the perifoveal regions probably indicate effects of lutein containing supplements. Eyes with early AMD showed no significant MPOD changes. The persistence of ring-like MPOD distributions over time seems to confirm previous reports suggesting that rings are mostly determined by anatomical structures.

## Additional file

**Additional file 1: Figure S1.** Box plots of the MPOD changes in quintiles, by eccentricity from the fovea.

## Abbreviations

ΔMPOD: changes of MPOD
AF: autofluorescence
AMD: age related macular degeneration
BMI: body mass index
D.U.: density units
MARS: Münster Ageing and Retina Study
MP: macular pigment
MPOD: macular pigment optical density
RPE: retinal pigment epithelium
