# Supplementary material for: Changes of macular pigment optical density in elderly eyes: a longitudinal analysis from the MARS study
Source: Int J Retina Vitreous. 2016 Jun 1;2:14. doi: 10.1186/s40942-016-0039-6 (PMC5088485; doi:10.1186/s40942-016-0039-6)
Supplement: Supplementary file 1 — Additional file 1: Figure S1. Box plots of the MPOD changes in quintiles, by eccentricity from the fovea. [file 40942_2016_39_MOESM1_ESM.docx]

0.4

0.2

0.0

-0.2

-0.4

.< <=-0.1

-0.1<

-0.02786<

0.05831<

>0.12107

<=-0.02786 <=0.05831 <=0.12107

OD_025 Quintiles

0.4

0.2

0.0

-0.2

-0.4

.< <=-0.07

-0.07<

<=-0.01498

-0.01498<

<=0.04

OD_05 Quintiles

0.04<

<=0.10491

>0.10491

0.4

0.2

0.0

-0.2

.< <=-0.06

-0.06< <=-0.01 -0.01< <=0.03 0.03< <=0.08

OD_10 Quintiles

>0.08

0.3

0.2

0.1

0.0

-0.1

.< <=-0.02

-0.02< <=0 0< <=0.02 0.02< <=0.05

OD_20 Quintiles

>0.05

OD_05 Change

OD_025 Change

OD_20 Change

OD_10 Change
